# *Katarium polorum* n. sp., n. g., a novel thecofilosean amoeba (Cercozoa, Rhizaria) from the polar oceans

**DOI:** 10.1111/jeu.13071

**Published:** 2024-11-29

**Authors:** Marcel Dominik Solbach, Michael Bonkowski, Kenneth Dumack

**Affiliations:** ^1^ Terrestrial Ecology, Institute of Zoology University of Cologne Cologne Germany

**Keywords:** Cercozoa, free‐living amoebae, marine, polar, Tectofilosida, Thecofilosea

## Abstract

Thecate amoebae play important roles in terrestrial and aquatic ecosystems. This study introduces a novel thecofilosean amoeba from Arctic and Antarctic sea sediments. Phylogenetic analysis based on the 18S rDNA sequence places it in the family Chlamydophryidae (order Tectofilosida, class Thecofilosea). However, the novel organism exhibits a significant genetic divergence and distinct morphology from its closest relatives, prompting us to erect the novel genus *Katarium* with its type species *Katarium polorum*. *K. polorum* is a consumer of diatoms and prokaryotes, indicating an important role in nutrient cycling in the polar marine food webs.

## INTRODUCTION

Shell‐bearing amoebae are globally distributed microorganisms, found across a wide range of habitats, where they play pivotal roles in nutrient cycling and microbial food webs by consumption of bacteria and other microeukaryotes. Shell‐bearing amoebae have independently evolved in several eukaryotic lineages, including the phylum Cercozoa (Cavalier‐Smith, 1998). Members of the Cercozoa are among the most dominant protists in terrestrial, freshwater, and marine ecosystems (Burki et al., 2021).

For a long time, many groups within Cercozoa were known only from their 18S rDNA sequences obtained from environmental sequencing studies and were therefore proposed as “Novel Clades” without any morphological information (Bass & Cavalier‐Smith, 2004). Relatively recently the marine Thecofilosea *Trachyrhizium urniformis* (Shiratori & Ishida, 2016) was described as a close relative of “Novel Clade 4,” and was soon accompanied by the freshwater species *Lecythium hyalinum*, *Lecythium jennyae* (Dumack, Mausbach, et al., 2017), and *Diaphoropodon archeri* (Dumack et al., 2018) based on molecular data. “Novel Clade 4” is since treated as equivalent to the family Chlamydophryidae (established by De Saedeleer (1934) as “Chlamydophryinae”), despite lacking molecular information on the eponymous genus *Chlamydophrys* (Belar, 1921). The Chlamydophryidae belong to the order Tectofilosida (Cavalier‐Smith & Chao, 2003) which comprises filose amoebae with a theca (i.e. organic “test”).

While thecofilosean amoebae are well studied in terrestrial and limnic ecosystems (see Dumack et al., 2018; Dumack, Baumann, & Bonkowski, 2016; Dumack, Flues, et al., 2017; Dumack, Mausbach, et al., 2017; Dumack, Müller, & Bonkowski, 2016; Öztoprak et al., 2020; Solbach et al., 2021), literature about marine amoeboid members of this group is scarce. Most marine Thecofilosea are reported to be flagellated, for example, *Cryothecomonas* (Drebes et al., 1996), *Protaspa* (Hoppenrath & Leander, 2006a), *Ebria* (Hoppenrath & Leander, 2006b), *Hermesinum* (Dumack et al., 2022), and *Ventrifissura* (Shiratori et al., 2020). Another prominent group of marine Thecofilosea are the Phaeodaria (or Phaeodarea), but they usually possess a siliceous skeleton (scleracoma), resulting in a very distinctive morphology (Nakamura & Suzuki, 2015). So far, there are only two marine members in the abovementioned amoeboid order Tectofilosida: *Trachyrhizium urniformis* (Shiratori & Ishida, 2016) and *Lecythium minutum* (De Saedeleer, 1934).

This is surprising since Thecofilosea often make up a large proportion of sequencing reads in marine environmental sequencing studies of Cercozoa (Cheng et al., 2022). The dominant thecofilosean taxa from studies conducted in the Arctic Ocean (Marquardt et al., 2016; Rapp et al., 2018; Sommeria‐Klein et al., 2021; Xu et al., 2020) or Antarctic Ocean (Rozema et al., 2017; Sommeria‐Klein et al., 2021) usually belong to the *Protaspa*/*Cryothecomonas* lineage (order Cryomonadida), which are flagellated consumers of diatoms or other unicellular eukaryotes. However, these studies also always retrieved a nonneglectable proportion of sequences belonging to undescribed Thecofilosea. These undetermined taxa are usually overlooked or ignored because no morphological or ecological information is available. It is not far‐fetched to assume that there are many undiscovered marine Thecofilosea with an amoeboid morphotype, potentially filling a similar ecological niche as their terrestrial and limnic counterparts.

In this study, we present a newly discovered thecofilosean amoeba found in sea sediment samples of both polar oceans (Arctic Ocean: Svalbard; Antarctic Ocean: Livingston Island). Sequencing of the 18S gene and morphological observations indicate that this amoeba is new to science. Hence, we erect the novel genus *Katarium* with its type species *Katarium polorum*.

## MATERIALS AND METHODS

### Sampling and cultivation

Sediment samples (approximately 1 cm depth) including sea water from the Arctic Ocean were collected at the coast in Ny‐Ålesund, Kongsfjorden, Svalbard (78.925796, 11.924552) in August 2022. Samples from the Antarctic Ocean were collected on the shoreline of the Byers Peninsula, Livingston Island, maritime Antarctica (−62.666946, −61.095524) in January 2023.

For several weeks, the samples were regularly screened for thecofilosean amoebae. Target cells were collected with a glass micropipette under a Nikon Eclipse TS100 inverted light microscope (up to 400x magnification, phase contrast) and transferred to F/8 medium (Guillard, 1975) in a 24‐well plate. The cells were repeatedly transferred to fresh F/8 medium until cultures free from other eukaryotes were obtained. In contrast to many other thecofilosean amoebae, the here obtained strains were observed to consume prokaryotes (presumably bacteria) next to eukaryotic prey. Two stable cultures, one from the Arctic Ocean (Svalbard, strain CCAP 1938/1), and one from the Antarctic Ocean (Livingston Island, strain CCAP 1938/2), could be established in F/8 medium without the addition of eukaryotic prey organisms. Instead, an autoclaved quinoa seed was added to each culture flask as an additional carbon source. Cultures were stored in 50 mL culture flasks at 4°C in the dark. Both strains were submitted to the Culture Collection of Algae and Protozoa (CCAP).

### Light microscopy

Cells were observed and photographed using a Nikon Eclipse 90i microscope (up to 1000X magnification, DIC) with a Nikon digital sight DS‐U2 camera (program: NIS‐Elements V4.13.04). Cell size was measured from photographs using ImageJ version 1.53k (Schneider et al., 2012). To obtain clearer photographs of the filopodia, culture flasks were incubated on ice for several minutes before imaging, as the filopodia were highly sensitive to heat and disintegrated rapidly when exposed to the microscope light.

For observations of diatom consumption, new culture flasks with fresh medium were prepared, and a different strain of diatoms, isolated from the same samples, was added to each flask. A total of six pennate diatom strains with varying sizes and morphologies were tested: *Cylindrotheca* sp. (Arctic, lanceolate), *Nitzschia* sp. (Arctic, sigmoid), *Navicula* sp. (Antarctic, fusiform), *Nitzschia* spp. (Antarctic, two strains with different sizes, fusiform), and *Haslea* sp. (Antarctic, fusiform). The cultures were incubated for 3 days, after which they were examined for the presence of food vacuoles containing diatom remnants.

### DNA extraction, amplification, and sequencing

DNA was extracted from 1 mL of each culture using the DNeasy Blood & Tissue Kit (Qiagen, Venlo, Netherlands) according to the manufacturer's instructions. Amplification of the 18S (SSU) rRNA gene was performed using the general eukaryotic primers EukA and EukB (Medlin et al., 1988). A volume of 1 μL of extracted DNA was added to 17 μL PCR mixture. The mixture contained 1.7 μL 10 μM primer EukA, 1.7 μL 10 μM primer EukB, 0.34 μL 10 mM dNTPs, 1.7 μL Dream Taq Green Buffer, 0.17μL DreamTaq polymerase (Thermo Fisher Scientific, Dreieich, Germany), and 11.4 μL water. The following PCR conditions were used: initial denaturation at 95°C for 5 min, 35 cycles (denaturation at 95°C for 30 s, annealing at 50°C for 30 s, elongation at 72°C for 2 min), terminal extension at 72°C for 7 min, and cooling at 10°C. A volume of 8 μL of the PCR products was purified by adding 0.15 μL of Exonuclease I, 0.9 μL of FastAP, and 1.95 μL of water, then heating at 37°C for 30 min, and subsequently at 85°C for 20 min. The purified PCR products were sequenced with the primers EukA, EukB, and the Cercozoa‐specific primers S615F_Cerco (Fiore‐Donno et al., 2020) and S947R_Cerco (Fiore‐Donno et al., 2018) using the BigDye Terminator Cycle Sequencing Kit (Thermo Fisher Scientific, Dreieich, Germany) and an ABI PRISM automatic sequencer at the Cologne Center for Genomics (CCG). The obtained chromatograms were manually checked for sequencing errors in Chromas V2.6.6 (Technelysium Pty. Ltd., Australia), and the partial sequences were assembled into sequence contigs in SeaView V4.6 (Gouy et al., 2010).

### Phylogenetic analysis

The two sequence contigs were added to an alignment of representative sequences belonging to all major lineages of the Cercozoa with a focus on Thecofilosea (Phaeodarea were deliberately excluded as they usually form a “long branching” clade). The alignment contained 114 sequences in total. The sequences were aligned in MAFFT using the L‐INS‐I algorithm V 7.221 (Katoh & Standley, 2013). The alignment was manually cut to 1582 selected sites. Phylogenetic trees were inferred in CIPRES (Miller et al., 2010) with the GTR + I + G model in RAxML (Stamatakis, 2014) with 200 random taxon additions for the maximum likelihood tree search and a 200 replicate bootstrap analysis, and Bayesian analysis in MrBayes (Altekar et al., 2004; Ronquist & Huelsenbeck, 2003). The Bayesian analysis was set up with sampling every 100 generations and diagnostics every 5000 generations, with 25% of the total 1.585 million generations discarded as burn‐in. Convergence was assessed using the average standard deviation of split frequencies (ASDSF), and the analysis was stopped when the ASDSF reached the threshold of 0.01. The results of the Bayesian analysis were added to the maximum likelihood tree, and the tree was visually adjusted in iTOL v6 (Letunic & Bork, 2024) and Adobe Illustrator CS6 (Adobe Inc., Delaware, USA).

## RESULTS AND DISCUSSION

**FIGURE 1:**
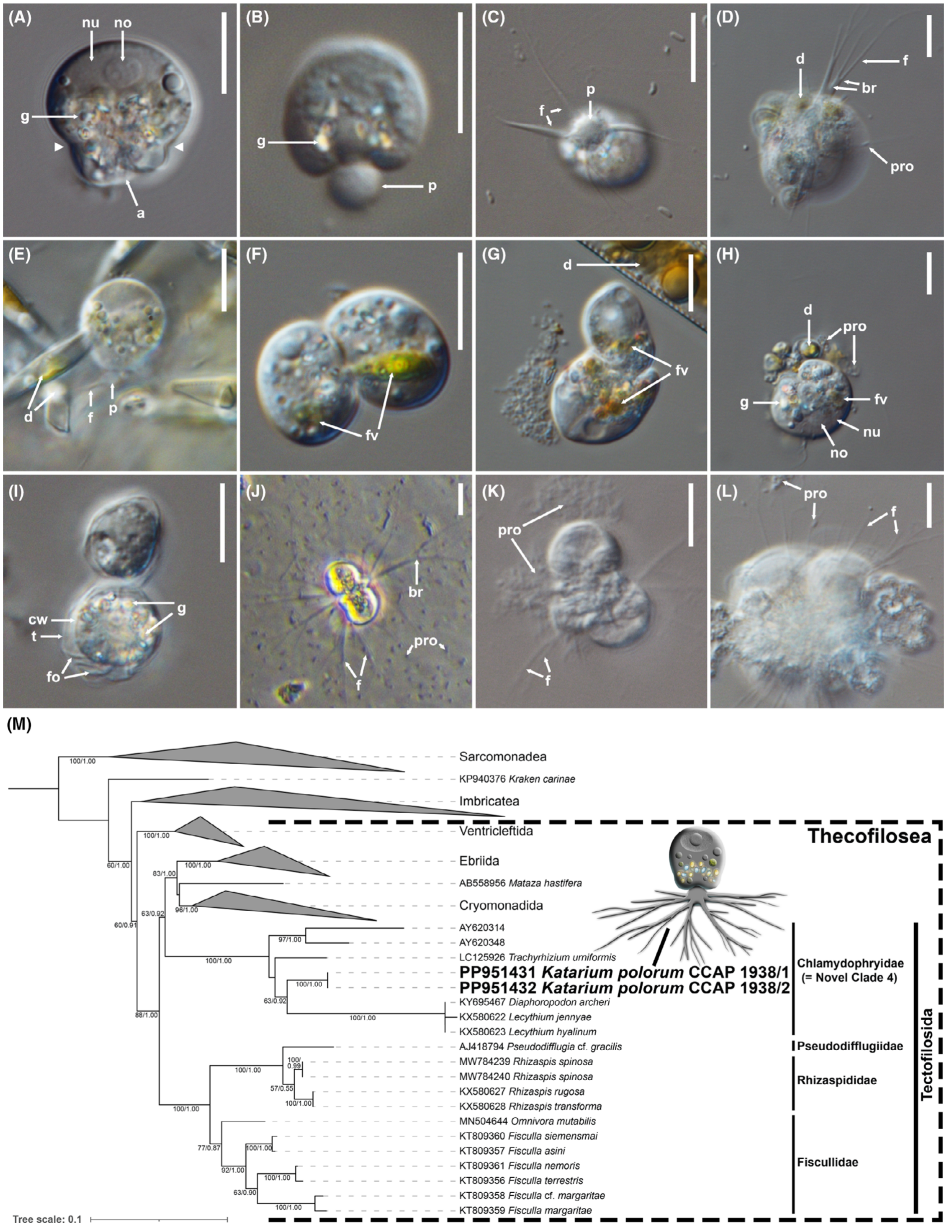
(A–L): Cellular features of *Katarium polorum* (type strain CCAP 1938/1) investigated by light microscopy. Scale bars indicate 10 μm. (A–J): Living cells from cultures. (K and L): Fixed cells from type material. (A): Average cell with visible oval, apically located nucleus and nucleolus and typical indentation of the theca above the aperture (arrowheads). (B): Individual with pronounced protrusion of cytoplasm. (C): Basal view of a cell with protruding cytoplasm from which the filopodia emerge. (D): Individual feeding on diatoms (small *Nitzschia* sp.) with pronounced branching filopodia. (E): Cells feeding on *Nitzschia* sp. (F): Cells with pronounced food vacuoles containing remnants of *Cylindrotheca* sp. (G): Cells with pronounced food vacuoles containing remnants of *Haslea* sp. (H): Cells with pronounced food vacuoles feeding on a small *NItzschia* sp. The same cell is displayed in D. (I): Dormant stages (cysts) of two cells. (J): Small aggregate/colony with fully displayed filopodia. Image taken with inverted microscope. (K): Small aggregate feeding on prokaryotes. (L): Larger aggregate with pronounced filopodia feeding on a debris particle of the culture medium with prokaryotes. a, aperture; br, branching filopdia; cw, cyst wall; d, diatom; f, filopodium; fo, fold; fv, food vacuole; g, layer of granules; p, protrusion of cytoplasm; pro, prokaryotes; t, theca. (M): Maximum likelihood SSU rDNA phylogenetic tree. Branches report the support values of maximum likelihood and Bayesian inference analysis (ML/BI). Support values below 50% bootstrap are omitted. Only the order Tectofilosida is expanded, and the remaining groups are collapsed. *Katarium polorum* is highlighted in bold and a schematic illustration is provided.

*Katarium polorum* n. sp., n. g., is a filose amoeba with an organic theca. The theca is roundish and slightly oblong. Single cells have a height of approximately 15.2 μm (± 2.8 μm, *n* = 40) and width of approximately 13.5 μm (± 2.5 μm, *n* = 40). Starving cells are usually wrinkled due to folds in their theca. Those folds often result in a slight indentation above the aperture (Figure 1A). The nucleus is oval and located at the apical end of the cell, with usually a single oval, or round nucleolus (Figure 1A). Each cell has a round aperture, from which often a part of the cytoplasm protrudes (Figure 1B,C,E). A protrusion of this kind was not reported for any other chlamydophryid and it seems to be a morphological peculiarity of *K. polorum*. Filopodia emerge from this protrusion (Figure 1C–E). The filopodia often branch (Figure 1D,J), but anastomosing and granules in the filopodia could not be observed. The filopodia were very sensitive to light/heat and quickly dissolved when observed under the microscope. Sometimes, cells were not attached to the surface but were floating in the culture medium with straight, extended filopodia as also described for *Lecythium hyalinum* (Dumack, Mausbach, et al., 2017). Cells in older cultures tend to form aggregates consisting of a few, or up to dozens of cells (Figure 1J–L). Cells display a zonation of the cytoplasm with a layer of highly reflective granules/crystals in the central horizontal plane (see especially Figure 1A,B,H,I) which seems to be characteristic for most Chlamydophryidae (compare Belar, 1921; Dumack, Mausbach, et al., 2017). Occasionally, “monstrosities” (large multinucleate cells) can be observed, usually as part of an aggregate. Cells in old cultures are usually inactive, that is, they are detached from the surface and float without extended filopodia, and sometimes form resting stages (cysts, Figure 1I). Even when stored at 4°C, cells of *Katarium polorum* were still highly active, that is, they were displaying filopodia, moving, feeding, and dividing. This implies that the species is well adapted to the low temperatures of the Arctic and Antarctic Oceans. *K. polorum* consumes diatoms (Figure 1E–H) like most other described Chlamydophryidae but can also consume bacteria (Figure 1D,K,L) like *Trachyrhizium urniformis* (Shiratori & Ishida, 2016).

As noted, *Katarium polorum* shares many similarities with members of the family Chlamydophryidae, while also displaying some unique features. The protruding cytoplasm of *K. polorum* is reminiscent of the vacuolated cytoplasm of *Lecythium hyalinum*, but lacking vacuoles. Both *L. hyalinum* and *L. jennyae* (Dumack, Mausbach, et al., 2017) are freshwater organisms and are notably larger than *K. polorum*. Another freshwater chlamydophrid, *Diaphoropodon archeri*, is also larger, with an elongated theca bearing distinct external rods and xenosomes (Dumack et al., 2018).

*Katarium polorum* is most similar to *Trachyrhizium urniformis* (Shiratori & Ishida, 2016). The species are of comparable size and shape, both inhabit marine environments, and both can consume bacteria. However, *T. urniformis* lacks the protruding cytoplasm and indentations of the theca seen in *K. polorum*, and its filopodia contain bidirectionally transported granules, which were not observed in *K. polorum*.

Besides *T. urniformis, Lecythium minutum* is the only other reported marine Tectofilosida, but *L. minutum* lacks molecular data. De Saedeleer (1934) described *L. minutum* from morphological observations of only two cells. He stated: “This form moves quickly using its pseudopodia: it is not excluded that we did not observe full unfolding of the pseudopodia since the organism was in constant motion” (translated from German). The described quick movement is atypical for thecofilosean amoebae and therefore seems to be the defining characteristic of *L. minutum*. Motion of this kind was not observed in our isolated strains. Furthermore, protruding cytoplasm as we observed for our isolates (Figure 1B,C,E) was not reported for *L. minutum*. We conclude that our newly isolated strains do not correspond to *L. minutum*, nor any other described chlamydophrid, but represent an undescribed organism.

The 18S gene sequence contigs of both strains were identical and had a length of 1710 nt. The sequences were submitted to NCBI GenBank (CCAP 1938/1: PP951431, CCAP 1938/2: PP951432). The sequences showed high similarity of 99.53% to an environmental sequence of marine samples from Isfjorden, West Svalbard (NCBI accession number KT816918; Marquardt et al., 2016). The closest hit of a described organism was *Trachyrhizium urniformis* (LC125926; Shiratori & Ishida, 2016), with a sequence similarity of a mere 94.7%. Also according to our phylogenetic analysis, *Katarium polorum* groups within the family Chlamydophryidae (= Novel Clade 4; order Tectofilosida, class Thecofilosea) (Figure 1M). While the family Chlamydophryidae is fully supported, the relationships within the family are not well resolved. Recent phylogenetic analyses are inconclusive about the monophyly of the order Tectofilosida (see Dumack, Mausbach, et al., 2017). In our study, the Chlamydophryidae group is next to Ebriida, Matazida, and Cryomonadida, but the support values are only moderate (Figure 1M). Even with the novel sequences, we cannot support or reject the monophyly of Tectofilosida.

The observation of *Katarium polorum* strains with identical 18S sequences from both polar oceans may seem surprising but is consistent with previous studies showing significant overlap in microbial communities between the Arctic and Antarctic. Wolf et al. (2015) found an average overlap of 11.2% in protist OTUs between the two oceans, and several studies have shown that bacterial communities in these regions are more similar to each other than to those in other oceans (Cao et al., 2020; Ghiglione et al., 2012; Kleinteich et al., 2017; Sul et al., 2013). Similar patterns have been observed for protists using isolation techniques, with identical 18S sequences reported for Foraminifera (Pawlowski et al., 2007) and acanthoecid choanoflagellates (Nitsche & Arndt, 2015) from both polar oceans. These studies imply that there is exchange of the polar marine microbiomes. The most likely distribution mechanisms are oceanic currents driven by thermohaline circulation (Cavicchioli, 2015), as well as potential vectors like migratory birds (e.g. the Arctic Tern; Egevang et al., 2010) or ballast water from ships (Galil & Hülsmann, 1997). Furthermore, it is plausible that the cysts of *K. polorum* (Figure 1I) are capable of long‐distance transport.

Based on the morphological and molecular considerations discussed above, we erect the novel genus *Katarium* with its type species *Katarium polorum*:

## TAXONOMIC ACTIONS

Taxonomic summary:

Phylum Cercozoa (Cavalier‐Smith, 1998)

Class Thecofilosea (Cavalier‐Smith, 2003)

Order Tectofilosida (Cavalier‐Smith & Chao, 2003)

Family Chlamydophryidae (De Saedeleer, 1934)

### *Katarium* Solbach, n. g.

Diagnosis: Marine filose amoebae with hyaline, organic theca, and round aperture from which a part of the cytoplasm protrudes. From this protrusion, the filopodia emerge. No granules in filopodia. Theca often has indentations above the aperture. Zonation of cells with a layer of highly reflective granules.

Type species: *Katarium polorum*.

Remarks: The genus *Katarium* is currently monotypic. For more details, refer to the following species description.

Etymology: cataracta, f (= waterfall) [Latin], −ium, neuter singular morphological suffix [Latin]. The genus name “*Katarium*” refers to the aquatic habitat of the type species. Furthermore, it is an allusion to the fictional character “Katara” in the animated series Avatar: The Last Airbender (created by Michael Dante DiMartino and Bryan Konietzko, Nickelodeon Animation Studio, USA). Katara is a “waterbender” from the Southern Water Tribe with relatives from the Northern Water Tribe. This refers to the fact that the type species *Katarium polorum* was found close to both the North and South Pole. *Katarium* is considered neuter, like most other described genera in the family Chlamydophryidae.

ZooBank LSID: urn:lsid:zoobank.org:pub:3832C860‐DD40‐42C7‐8E9C‐0C4FFB6D2413, nomenclatural act:urn:lsid:zoobank.org:act:ED18097A‐8626‐4132‐AED0‐A0943EE97D86.

### *Katarium polorum* Solbach, n. sp.

Diagnosis: Marine filose amoeba with hyaline, organic theca. Cells are roundish and slightly oblong, approximately 15 μm in size. Oval nucleus located at the apical end of the cell, with usually a single nucleolus. Round aperture, from which a part of the cytoplasm protrudes. From this protrusion, filopodia emerge. Filopodia are usually long, thin, and branching. Anastomosing and granules in the filopodia were not observed. Theca often has indentations above the aperture. Zonation of cells with a layer of highly reflective granules in the central horizontal plane. So far only isolated from Polar Regions, and appears to be psychrophilic (adapted to cold). Consumes prokaryotes and diatoms.

Etymology: polus, m (= pole), Genitive Plural: polorum (= the poles') [Latin]. The species name refers to the fact that *Katarium polorum* was found close to both geographic poles (North and South Pole).

Type‐generating strain: Strain CCAP 1938/1 is deposited in the Culture Collection of Algae and Protozoa (CCAP).

Sequence of type generating strain (18S SSU rDNA): PP951431.

Type material (hapantotype): A glass slide with fixed individuals (85% glycerol, 15% phosphate‐buffered saline) of the type strain CCAP 1938/1 is deposited in the zoological collection of the Institute of Zoology, University of Cologne. This material constitutes the name‐bearing type of this species.

Photographs of type generating strain: See Figure 1 (Figure 1K,L display cells of the hapantotype).

Illustrations of type generating strain: See Figure 1M.

Type locality: Marine sediment of the coast in Ny‐Ålesund, Svalbard (78.925796, 11.924552).

ZooBank LSID: urn:lsid:zoobank.org:pub:3832C860‐DD40‐42C7‐8E9C‐0C4FFB6D2413, nomenclatural act: urn:lsid:zoobank.org:act:DFA78522‐99D5‐48FA‐A0BB‐FC30B649DA88.
